# Modeled energetics of bacterial communities in ancient subzero brines

**DOI:** 10.3389/fmicb.2023.1206641

**Published:** 2023-07-26

**Authors:** Georges Kanaan, Tori M. Hoehler, Go Iwahana, Jody W. Deming

**Affiliations:** ^1^School of Oceanography and Astrobiology Program, University of Washington, Seattle, WA, United States; ^2^NASA Ames Research Center, Moffett Field, CA, United States; ^3^International Arctic Research Center, University of Alaska Fairbanks, Fairbanks, AK, United States

**Keywords:** cryopeg, Arctic, extremophiles, permafrost, maintenance energy

## Abstract

Cryopeg brines are isolated volumes of hypersaline water in subzero permafrost. The cryopeg system at Utqiaġvik, Alaska, is estimated to date back to 40 ka BP or earlier, a remnant of a late Pleistocene Ocean. Surprisingly, the cryopeg brines contain high concentrations of organic carbon, including extracellular polysaccharides, and high densities of bacteria. How can these physiologically extreme, old, and geologically isolated systems support such an ecosystem? This study addresses this question by examining the energetics of the Utqiaġvik cryopeg brine ecosystem. Using literature-derived assumptions and new measurements on archived borehole materials, we first estimated the quantity of organic carbon when the system formed. We then considered two bacterial growth trajectories to calculate the lower and upper bounds of the cell-specific metabolic rate of these communities. These bounds represent the first community estimates of metabolic rate in a subzero hypersaline environment. To assess the plausibility of the different growth trajectories, we developed a model of the organic carbon cycle and applied it to three borehole scenarios. We also used dissolved inorganic carbon and nitrogen measurements to independently estimate the metabolic rate. The model reconstructs the growth trajectory of the microbial community and predicts the present-day cell density and organic carbon content. Model input included measured rates of the *in-situ* enzymatic conversion of particulate to dissolved organic carbon under subzero brine conditions. A sensitivity analysis of model parameters was performed, revealing an interplay between growth rate, cell-specific metabolic rate, and extracellular enzyme activity. This approach allowed us to identify plausible growth trajectories consistent with the observed bacterial densities in the cryopeg brines. We found that the cell-specific metabolic rate in this system is relatively high compared to marine sediments. We attribute this finding to the need to invest energy in the production of extracellular enzymes, for generating bioavailable carbon from particulate organic carbon, and the production of extracellular polysaccharides for cryoprotection and osmoprotection. These results may be relevant to other isolated systems in the polar regions of Earth and to possible ice-bound brines on worlds such as Europa, Enceladus, and Mars.

## 1. Introduction

On Earth, bacteria often encounter energy-limited environments. Their prevalent physiological state is understood to be energy-limited (Lever et al., 2015). For example, the vast subsurface biosphere is energy-limited (Jørgensen and Boetius, 2007), yet sustains abundant microbial life (Teske, 2005; Jørgensen and Marshall, 2016). Understanding the strategies that allow bacteria to survive in such extreme environments prompts the question: what is their minimum metabolic requirement? Here we investigate the energetic needs over time of bacterial communities in cryopeg brine, a subzero hypersaline environment geologically isolated from surface inputs. We seek to answer the question posed by estimating the cell-specific metabolic rates of the bacterial communities residing in these extreme settings.

Cryopeg brines are considered extreme for various reasons, one of which is their assumed energetic isolation. These brines are volumes of hypersaline subzero liquid water found in permafrost well below the surface. The Utqiaġvik system of cryopegs in the high Alaskan Arctic at approximately 8 m below surface has a temperature around −6°C and total salt concentration around 120 ppt (Cooper et al., 2019). Carbon-14 (^14^C) measurements suggest the brines have been enclosed for approximately 40 ka BP (Iwahana et al., 2021). Two types of cryopeg brines exist within the permafrost here: those encased by a layer of frozen marine sediments and those encased by massive ice. Both types are thought to be isolated hydrologically (Iwahana et al., 2021). Together, these properties describe an environment that is not only energy-limited, but also energetically costly to inhabit due to challenging conditions. Despite these conditions, cell densities range from 10^5^ to 10^8^ cells mL^−1^ brine (Cooper et al., 2019), comparable to previously sampled cryopeg brines across the Arctic (Gilichinsky et al., 2003, 2005; Bakermans et al., 2006). Metagenomic analyses show the presence of overwhelmingly heterotrophic bacterial communities dependent on organic carbon for their source of energy (Rapp et al., 2021; Cooper et al., 2022).

Investigations of cryopeg systems are relevant not only to our understanding of Earth-bound ecosystems but, excitingly, can inform our understanding of possible extraterrestrial life. Life within the icy mantles of Europa or Enceladus, and possibly the subsurface of Mars, could be inhabiting similarly extreme, energy-limited environments (Marion et al., 2003; Priscu and Hand, 2012; Sholes et al., 2019; Gomez-Buckley et al., 2022). To our knowledge, no estimate of cell-specific metabolic rate in subzero hypersaline environments is available. The objective of this study was to develop such estimates and contribute to our understanding of the habitability of subsurface subzero brines, be they Earth-bound or extraterrestrial.

We considered that the key to understanding the Utqiaġvik cryopeg system was to reconcile high cell densities with potential microbial kinetics and the available energy pool. We hypothesized that the minimum metabolic rate of the brine residents would be relatively high due to the extreme conditions and corresponding need to synthesize protective compounds, making the requirement for organic carbon correspondingly high to account for the observed cell densities. To test this hypothesis, we first made a series of simplifying assumptions to enable a first-order analysis of the system. The objective was to estimate the cell-specific metabolic rate of a resident community, assuming organic carbon as the sole energy source and considering two microbial growth trajectories to provide an upper and lower bound of this rate. This approach required us to measure the quantity of organic carbon in the sediments surrounding the brines. We then constructed a model of the organic carbon cycle within the brine, which allowed us to reconstruct microbial growth trajectories and relate them to available organic carbon. A sensitivity analysis of model parameters was performed to understand their relevance to model results and thus the limitations of our model. These parameters included the enzymatic conversion of particulate organic carbon (POC) to dissolved organic carbon (DOC) in the brine. POC represents the dominant form of organic carbon in surrounding permafrost yet is not available to bacteria until hydrolyzed to smaller molecular weight compounds (DOC).

Finally, we compared the model predictions to the available observations, which together allowed us to propose the system’s microbial history under energetic isolation. We produced plausible simulations hinging on the precision of key parameters, and thus could identify research areas that would further advance understanding of bacterial energetics in extreme environments.

## 2. Physical and biological context for the model

To provide context for our model we outline the relevant environmental characteristics of cryopeg brines in this section. The physical characteristics guided the development of the equations governing environmental interactions in the model. Microbial energetics of the bacteria found in the cryopeg brine constrained our analyses and provided an understanding of the biology that the model attempts to resolve. Together, the physics and biology of the cryopeg brine underlie the design of our work, and the thoughts behind the analyses conducted.

### 2.1. Physical characteristics of cryopeg brines

Cryopeg brines are volumes of hypersaline water that occur in cryopeg, a basal layer in permafrost of unfrozen sediment perennially at subzero temperatures (van Everdingen, 2005). The brines studied herein were collected from below the Barrow Permafrost Tunnel at a depth of approximately 8 m below the surface, on the northernmost coast of Alaska. They are thought to have formed from saturated marine sediments in a lagoonal environment (Iwahana et al., 2021). As sea level fell during glaciation, these sediments would have been exposed to the atmosphere and become desiccated, causing previously dissolved solutes to concentrate and, following entrainment into permafrost, depress the freezing point of water to yield brine (Gilichinsky et al., 2003, 2005; Iwahana et al., 2021). This cryoconcentration effect leads to high salinities and possibly to concentrated organic matter. The marine origin of these brines is supported by ionic and microbiological evidence (Colangelo-Lillis et al., 2016; Iwahana et al., 2021). The temperature of these Alaskan brines perennially falls within a narrow range of −6 to −8°C and is thought to have remained within this range over their lifespan (Colangelo-Lillis et al., 2016; Cooper et al., 2019; Iwahana et al., 2021; Osman et al., 2021). The salinity of these brines ranges from 109 to 140‰ salt (Cooper et al., 2019). These brines are hydrologically isolated from each other as evidenced by the different pressure heads and equilibrium brine levels (Iwahana et al., 2021). Moreover, the combination of ice content plugging sediment pores and bacteria attached to surfaces is thought to preclude input of cells into the brine from the surrounding environment (Gilichinsky et al., 2005). However, possible input during partial melting at the brine/ice boundary cannot be excluded given slight seasonal temperature oscillations. They may be relevant to the observed microbiological similarities between proximate massive ice and sediment brines (Cooper et al., 2019).

The Utqiaġvik cryopeg system presents brines encased by marine sediments, as previously observed in other cryopegs, as well as those newly discovered to be encased by massive ice, respectively called intra-sediment and intra-ice brines (Iwahana et al., 2021). Intra-ice brines are thought to have migrated along the temperature gradient into the massive ice around 11 ka BP. This migration is suggested by the equal age of organic carbon in the brine and the massive ice surrounding it (Iwahana et al., 2021).

**Figure 1:**
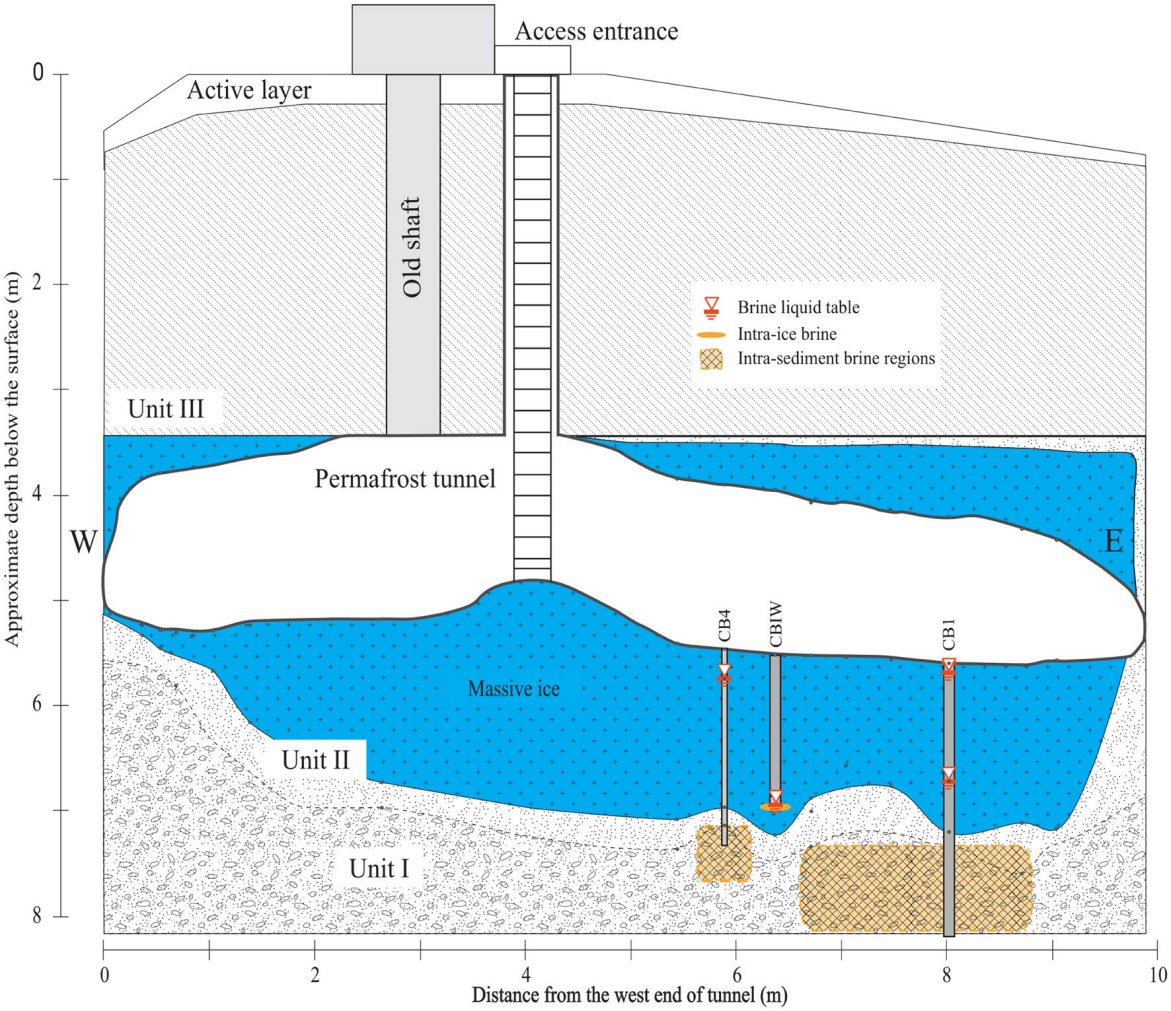
NNW-facing cross-sectional diagram of the Barrow Permafrost Tunnel providing access to cryopeg brines near Utqiaġvik, Alaska. Depicted are the cryopeg boreholes CB1, CBIW and CB4 considered in this study. CB1 and CB4 accessed intra-sediment brines 7–8 m below the massive ice; CBIW accessed intra-ice brine thought to have migrated upwards into the massive ice 11,000 years BP (Iwahana et al., 2021). Units I-III refer to permafrost regions (Meyer et al., 2010). Figure adapted from Iwahana et al. (2021).

We considered brines sampled from three distinct cryopeg boreholes in the Barrow Permafrost Tunnel (Figure 1): two intra-sediment brines from boreholes CB1 and CB4, and one intra-ice brine from borehole CBIW. CBIW was sampled twice, in 2017 and 2018. All brines were sampled in May and were at −6°C when sampled, with salinities of 115, 121 and 140‰ salt, respectively (Cooper et al., 2019). No *in situ* oxygen measurements have been made in these or other tunnel boreholes, but anaerobic conditions are expected within the brines (Iwahana et al., 2021).

Cryopeg brines considered here featured POC concentrations of 2–12 mM and DOC concentrations of 30–102 mM, which are high when compared to the typical micromolar concentrations in seawater (Mathis et al., 2005; Goñi et al., 2021). The DOC concentrations are also high when compared to porewaters of nearby (unfrozen) marine sediments (e.g., < 7 mM on the Alaskan north slope; Coffin et al., 2017) and other Arctic marine sediments (1–6 mM, Arnosti and Jørgensen, 2006; < 1 mM, Rossel et al., 2020). Measured inorganic nutrients were also relatively high: nitrate, nitrite, and phosphate were present in micromolar concentrations, while ammonium concentrations were at millimolar levels (Cooper et al., 2019). Sulfate concentrations were similar to those in Chukchi Sea water, potentially drawn down in the brines from more concentrated values due to sulfate reduction or to mirabilite formation (Marion et al., 1999; Iwahana et al., 2021).

### 2.2. Microbial energetics of cryopeg brines

Across the Arctic, cryopeg brines have been found to harbor sizeable microbial communities, ranging between 10^5^ and 10^8^ cells mL^−1^ (Gilichinsky et al., 2003, 2005; Cooper et al., 2019). These brine communities are composed of a diverse set of organisms, which can include species of *Marinobacter*, *Psychrobacter*, *Gillisia*, *Frigoribacterium*, *Rhodococcus, Polaribacter*, and *Sulfurospirillum* (Bakermans et al., 2003, 2006; Bakermans and Nealson, 2004; Gilichinsky et al., 2005; Colangelo-Lillis et al., 2016; Cooper et al., 2019). Community composition appears to differ between Arctic regions, but the use of different methods across studies limits this assessment. The cryopeg brines below the Barrow Permafrost Tunnel, however, have been the subject of in-depth microbiological characterization.

The dominant bacterium in the brines from CB1 and CBIW, two of the brines we considered in this study, was a novel species of *Marinobacter*, recently brought into culture (Cooper et al., 2022). On average it comprised 49% of the total community (Cooper et al., 2019). In the brine from CB4, this *Marinobacter* sp. was abundant, but the dominant bacterium was *Psychrobacter* sp. at 54% of the total community (Cooper et al., 2019). Model parameters for microbial community kinetics were therefore based on the available data for these organisms. Regarding bacterial densities by epifluorescence microscopy, the brines from CB1, CB4 and CBIW (averaged over both sampling years) harbored 5.70 × 10^6^, 1.14 ×10^7^ and 1.30 ×10^8^ cells mL^−1^, respectively, with dividing cells observed in all three brines (Cooper et al., 2019). Dissolved extracellular polysaccharides (dEPS) made up between 19 and 28% of the DOC pool. Particulate extracellular polysaccharides (pEPS) made up between 2 and 13% of the POC pool (Colangelo-Lillis et al., 2016; Cooper et al., 2019).

Organisms reliant upon a range of metabolisms have been detected in cryopeg brines, including heterotrophs, sulfate reducers, acetogens and methanogens (Gilichinsky et al., 2003, 2005; Cooper et al., 2019). The genus *Marinobacter* features a highly versatile set of metabolisms, allowing its members to inhabit a wide diversity of environmental niches. Species of this genus, including from cryopeg brines, possess a complete tricarboxylic acid (TCA) cycle and glyoxylate shunt. The glyoxylate shunt bypasses the production of carbon dioxide and may play a role in oxidative stress response (Ahn et al., 2017). A genomic analysis of four strains of the dominant *Marinobacter* sp. isolated from Utqiaġvik cryopeg brines further reveals its metabolic potential (Cooper et al., 2022). This *Marinobacter sp.* possesses the genes required to derive energy from a wide range of organic compounds, including all 20 amino acids (with the possible exception of asparagine). As a substitute for glycolysis, it encodes the Entner–Doudoroff pathway, considered an adaptation for energy efficiency at low temperatures (Czajka et al., 2018). Its pathways for nitrogen cycling include nitrate oxidation and reduction, dissimilatory nitrate reduction, nitric oxide reduction, and nitrous oxide reduction. A C-P lyase system encoded in its genome may facilitate the scavenging of phosphate from organophosphates. The variety of nitrogen, sulfur and metal-based redox reactions encoded in its genome supports a facultative anaerobic lifestyle.

For a heterotrophic *Psychrobacter* sp. isolated from Siberian cryopeg, Bakermans et al. (2003) measured resazurin reduction rate (as a proxy for respiration) and growth rate across a temperature range of −10 to 22°C. From ratios of these rates, they concluded that cell metabolic requirements increase substantially at subzero temperatures. Such an increase is unexpected when considering that base energetic needs scale predominantly as a function of temperature, increasing with warming (Tijhuis et al., 1993; Price and Sowers, 2004). Given the subzero temperature of cryopeg brines, a relatively low cell-specific metabolic rate would be reasonable to expect. However, multiple constraints may impose a higher energetic cost to life in this extreme brine system.

Constrained habitat volume and high cell density in cryopeg brines lead to higher rates of cell-to-cell (and virus-to-cell) contact than occur in seawater. Increased cell-to-cell contact rates exacerbate resource and space competition. An analysis of cryopeg brine metagenomes found high abundance of cells associated with the type VI secretion system and microcin C, both tools to lyse neighboring competitors (Rapp et al., 2021). This microbial weaponry can be understood within the framework of an energetic arms race. Such competition-associated costs could raise the energetic cost of living in this system.

Moreover, cryopeg brines host abundant viral communities of marine origin that appear to have mediated the exchange of genetic information (Colangelo-Lillis et al., 2016; Zhong et al., 2020). Viral interactions contribute to the rate of cell death by lysis, influencing the organic carbon cycle through release of cellular carbon content (Showalter, 2020). Thus, we can hypothesize a high turnover of cell biomass leading to increased energetic cost to maintain a steady-state population.

The required production of certain compounds may also increase the energetic cost of life in this system. Subzero brines are relatively viscous (Cox and Weeks, 1975), and must be especially viscous when they contain millimolar concentrations of EPS (Cooper et al., 2019), known for cryoprotection and osmoprotection (Marx et al., 2009; Deming and Young, 2017). Viscosity may lead to the creation of microscale environmental niches, for example, as cell lysis alters local biochemistry. Metagenomics on cryopeg brines revealed the abundance of genes encoding two-component signaling systems (Rapp et al., 2021). These systems would allow a bacterium to respond to a changing energetic landscape more rapidly to outcompete its immediate neighbors (Rapp et al., 2021). A microbial response to localized patches of high molecular weight organic matter may take the form of extracellular enzyme production. Extracellular enzyme activity (EEA) has been measured in cryopeg brines (Showalter, 2020), providing evidence of proactive management of substrate concentrations by the bacterial community. The production of extracellular compounds, from EPS to enzymes, is an additional energetic cost.

**Figure 2:**
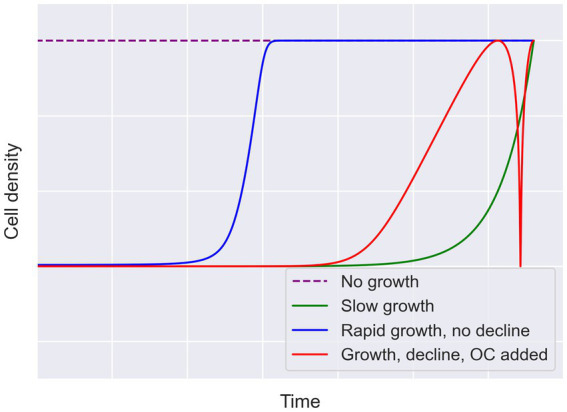
Hypothetical growth trajectories to account for a given endpoint cell density. Four possible cases for growth are illustrated here to describe why endpoint measurements leave open many possible trajectories. The case for rapid initial growth followed by stasis is presented in blue. The case for slow growth leading to the observed cell density is presented in green. In red, an intermediate growth rate, followed by decline due to insufficient energy and then an input of organic carbon (OC), depicts recovery of the community to the given endpoint. The no growth scenario, requiring the starting community to be as dense as the endpoint, is indicated by the dashed purple line. Growth lines are intended to be logarithmic, but all lines are conceptual (not drawn to scale).

Although the current genomic data available provide promising insights into the metabolisms supported by cryopeg brines, they do not allow us to determine whether these brines have supported energetically isolated microbial communities for 40,000 years. To understand the energetics of the Utqiaġvik brines, their energetic histories need to be reconstructed. This effort necessarily involves the reconstruction of the microbial growth trajectory over the lifespan of the brine. Figure 2 illustrates several possible growth trajectories. The different fluctuations or constancy in cell densities over time in each case lead to different energetic requirements. Simplistically, a community that spent most of its history at 10^5^ cells mL^−1^ will not require the same amount of energy as one at 10^8^ cells mL^−1^. More complex trajectories could also have occurred, such as when a microbial community declines due to insufficient energy, then recovers after an energy input (e.g., when intra-sediment brine is assumed to have migrated into massive ice).

## 3. Materials and methods

Here we indicate the methods used to quantify key variables, such as organic carbon, and show the equations developed to model the different processes involved in microbial energetics. Along with the mathematical outline of our model, we also present fundamental assumptions and limitations. All of the code used to describe and calculate the equations below and plot the results shown is available online on GitHub. The contents of this paper were generated from the most recent commit on the “paper” branch. Python v3.9 and Julia v1.8.2 (Bezanson et al., 2017) are used throughout, and plots were generated using Matplotlib v3.6.2 and Seaborn v0.12.1 (Hunter, 2007; Waskom, 2021).

### 3.1. Organic carbon and nitrogen in sediment and massive ice

To determine the POC content of sediments surrounding the cryopeg brines, we used the method of Verardo et al. (1990). Duplicate samples of approximately 20 g of material were cleanly removed from previously collected sediments (Iwahana et al., 2021) that had been stored frozen (−20°C) until analysis. In each case, the sediment material was placed in a 50-cc polypropylene tube, weighed, dried in an oven at 60°C for at least 48 h, and weighed again to obtain dry weight. Samples were then homogenized to a fine powder in a ceramic mortar and pestle and kept in the drying oven until further processing. In the Marine Chemistry Lab (School of Oceanography, University of Washington), 4–6 mg of each sample were weighed in duplicate in combusted silver “boats” after having been fumed in HCl for 24 h to remove inorganic carbonates. Samples were dried again in the 60°C oven while acetanilide standards were prepared. Before analysis, the samples were cooled in a desiccator for at least 24 h. The sediment organic carbon and nitrogen content was measured using a Model 440 CHN/O/S Elemental Analyzer and a combustion temperature of 1,050°C. The organic carbon and nitrogen content of massive ice from this site was already available (Colangelo-Lillis et al., 2016).

To convert our sediment carbon measurements to *in-situ* concentrations we assumed a dry sediment density of 2.625 g mL^−1^, the average of the density of kaolinite and sand. This assumption is guided by the fact that nearby sediment is composed of undefined clay minerals and sand (Iwahana et al., 2021). An expansion factor was needed to account for the fact that carbon quantification methods require thawing porewater, and that water density changes between its solid and liquid phases. We used a common expansion factor of 9.05% for permafrost porewater, and 8.042% for massive ice. Values for the volumetric ice content of permafrost, needed to determine the density of sediment *in situ*, were 73.1% in both CB1 and CBIW sediment, and 52.7% in CB4 sediment.

To determine DOC of porewater in the frozen sediment surrounding the cryopeg brines, subsamples of the sediment and ice were shaved off core sections with a sterile scalpel onto sterile aluminum foil. These subsamples were placed in 50-cc polypropylene tubes, weighed, and allowed to thaw at 4°C for 15 min before centrifuging for 10 min at 2,000 rpm in a benchtop centrifuge (IEC, Model HN-SII IM201) at 2°C. The resulting supernatant was decanted and filtered into a clean EPA vial using a 0.2-μm syringe filter, then frozen until analyzed. The tube of leftover sediment was reweighed for porewater/sediment normalization. DOC was measured using a Shimadzu TOC-VCSH DOC analyzer according to standard protocols in the Marine Chemistry Lab (School of Oceanography, University of Washington). In the case of massive ice, we lacked suitable samples for DOC analysis, so measurements of dEPS (Colangelo-Lillis et al., 2016) were used as a proxy for DOC.

### 3.2. Dissolved inorganic carbon

Dissolved inorganic carbon (DIC) was measured for a sample of CBIW brine taken in 2017 as an auxiliary to the ^14^C dating procedure (Iwahana et al., 2021). Brine sample of approximately 200 mL was degassed under vacuum by pumping to the 10^−4^ Torr range. Dissolved carbon dioxide was liberated by the addition of anhydrous phosphoric acid. Purified carbon dioxide was isolated from other gases by gas distillation. DIC was then measured by the University of Arizona, Accelerator Mass Spectrometry Lab.

### 3.3. Modeling scenarios

We considered three separate scenarios for modeling purposes. Each scenario is based on a unique occurrence of cryopeg brine below the permafrost tunnel. The scenarios are named after the borehole that yielded the brine they describe: CB1, CB4, and CBIW. The following variables define a scenario: brine cell density, brine POC and DOC concentration, surrounding POC and DOC concentration, post-enclosure carbon addition, bacterial growth rate, and carbon content per cell.

The CB1 scenario was chosen to represent the multiple intra-sediment brines in the Utqiaġvik region of common geology and microbial composition. It was paired with organic carbon measurements made on regional permafrost from the nearby Barrow Environmental Observatory (BEO). As the dominant organism in this brine was the newly isolated *Marinobacter* sp., its growth rate and a literature-informed approximation of its carbon content were used to represent the microbial community.

The CB4 scenario describes an intra-sediment brine that differs from the others in terms of its dominant organism, *Psychrobacter* sp. The published growth rate and carbon content for Arctic *Psychrobacter* sp. (Bakermans et al., 2003, 2006) were used to represent the microbial community. In this scenario we used our measurements of organic carbon in sediment immediately surrounding this brine.

The CBIW scenario represents cryopeg brine encased in massive ice instead of sediment. This brine is thought to have originated as intra-sediment brine, then migrated upwards into massive ice around 11,000 years BP to become surrounded by ice. This ^14^C-dating provided evidence of a possible mechanism for addition of organic carbon from the ice into the brine (Iwahana et al., 2021). The CBIW scenario is therefore the only one for which we have modeled an addition of organic carbon past the initial stage of brine enclosure. We used a lower bound estimate of the amount of carbon added, based on the amount of carbon in the massive ice currently surrounding the brine (Colangelo-Lillis et al., 2016). As CBIW brine was dominated by the new *Marinobacter* sp., we used its traits to represent the microbial community.

Specific values for the variables we used to define a model scenario are provided in Table 1. For scenarios CB1 and CBIW, where *Marinobacter* sp. dominated the brine community, the cell carbon content, $\alpha_{D},$ was taken from an average value for Arctic sea-ice bacteria from Nguyen and Maranger (2011), the closest relevant estimates for a subzero brine environment that we could find. For the CB4 scenario we took the average size for a proxy of its dominant bacterium, *Psychrobacter sp.*, as 0.365 μ m^3^ (Bakermans et al., 2006), using 148 fg C μ m^−3^ as the carbon conversion factor (Kirchman et al., 2009). The maximum growth rate, $\mu_{\max}$ , for scenarios CB1 and CBIW was derived from lab experiments with the *Marinobacter* sp. isolated from these cryopeg brines. The cultures were grown in nutrient-replete media under *in-situ* temperature and salinity (Cooper et al., 2022). For the CB4 scenario, we used the growth rate determined for a culture of *Psychrobacter sp.* grown in nutrient-replete media at −10°C (Bakermans et al., 2003). The model assumes these rates to be the *in-situ* growth rates, as they are the closest approximations available. We note that they were determined not only under nutrient-replete conditions but also under aerobic culture conditions, and therefore likely represent over-estimations of the *in-situ* growth rates given that cryopeg brine is assumed to be an anaerobic environment.

**Table 1:** Variables and values used to define each of three cryopeg brine scenarios.

Variable	Symbol (units)	CB1 scenario (source^a^)	CB4 scenario (source^a^)	CBIW scenario (source^a^)
Cell density	$N_{f}$ (cells mL^−1^)	5.70 × 10^6^ (CB1; Colangelo-Lillis et al., 2016)	1.14 × 10^7^ (CB4; Cooper et al., 2019)	1.39 × 10^8^ (CBIW; Cooper et al., 2019)
Sediment POC	$P_{0}$ (fg C cm^−3^)	1.64 × 10^13^ (BEO; this study)	1.75 × 10^13^ (CB4; this study)	1.64 × 10^13^ (BEO; this study)
Sediment DOC	$D_{0}$ (fg C cm^−3^)	3.41 × 10^10^ (BEO; this study)	6.17 × 10^11^ (CB4; this study)	3.41 × 10^10^ (BEO; this study)
Brine POC	$P_{f}$ (fg C mL^−1^)	1.49 × 10^11^ (CB1; Cooper et al., 2019)	4.97 × 10^10^ (CB4; Cooper et al., 2019)	2.38 × 10^10^ (CBIW; Cooper et al., 2019)
Brine DOC	$D_{f}$ (fg C mL^−1^)	1.23 × 10^12^ (CB1; Cooper et al., 2019)	1.02 × 10^12^ (CB4; Cooper et al., 2019)	3.60 × 10^11^ (CBIW; Cooper et al., 2019)
Added DOC^b^	$D_{in}$ (fg C mL^−1^)	0	0	3.88 × 10^10^ (massive ice; Colangelo-Lillis et al., 2016)
Added POC^b^	$P_{in}$ (fg C mL^−1^)	0	0	1.86 × 10^10^ (massive ice; Colangelo-Lillis et al., 2016)
Cell carbon content	$\alpha_{D}$ (fg C cell^−1^)	15.7 (Nguyen and Maranger, 2011)	54.04 (Bakermans et al., 2006; Kirchman et al., 2009)	15.7 (Nguyen and Maranger, 2011)
Net growth rate^c^	$\mu_{\max}$ (day^−1^)	0.06 (Cooper, 2021)	0.016 (Bakermans et al., 2003)	0.06 (Cooper, 2021)

^a^CB1, CB4, and CBIW indicate boreholes; BEO indicates Barrow Environmental Observatory. CB1 and CBIW were dominated by *Marinobacter* sp.; CB4, by *Psychrobacter* sp. ^b^Single addition at 29,000 years (11,000 years BP) into the 40,000-year trajectory. ^c^Net (community) growth rate was set to the maximum growth rate of the dominant bacterium (see Section 3.7).

### 3.4. Cell-specific metabolic rate

A primary goal was to develop an equation to estimate the cell-specific metabolic rate of the microbial community, which is described below (Equation 1). The cell-specific metabolic rate term was inspired by Pirt (1982), who proposed a constant base maintenance energy term and a second growth-dependent term without distinguishing different types of metabolisms. In our model, we have abstracted the total organic carbon (TOC) consumed to a cell-specific metabolic rate term, as we do not have a good approximation of the growth-dependent term. Metabolic rate as a function of growth rate would be needed to more accurately approximate the growth-dependent term. We account for organic carbon usage for biomass production (growth) separately.

We assumed that the brine upon initial enclosure contained POC and DOC pools equivalent to those present in the continuously frozen material currently surrounding the brines. In the CBIW scenario, we considered the surroundings to have been equal in TOC content to regional frozen sediment at depth in the permafrost (sampled at BEO). We then took the difference between starting TOC and TOC measured in the brines. This calculation accounted for any carbon addition during the 40,000-year period by adding it to the starting TOC quantity. We subtracted from this difference the quantity of organic carbon diverted toward biomass.

To obtain bounds for our approximation we considered two cases of cell growth. The lower bound case requires the available quantity of organic carbon to be divided among as many cells as possible to minimize this per-cell quantity. This case corresponds to one in which no growth occurs (Pirt, 1982). The upper bound case minimizes the cell quantity to maximize the cell-specific metabolic rate. This approach is intuitive by realizing that biomass is the only sink of carbon in this equation. Therefore, the upper bound case is represented by the slowest possible rate of exponential growth.

To calculate the slowest possible rate of exponential growth, we determined the growth rate of a community over the simulation time of 40,000 years by fitting an exponential function between the starting cell density of 10^5^ cells mL^−1^ and the observed ending cell density. The resulting rate is called the “minimum growth rate.” This approach assumes that the microbial community was growing over the entire lifespan of the enclosed brine, and hence does not allow for death or dormancy. Inherent to Equation 1 is that our system is feasible under the minimum growth rate with either cell-specific metabolic rate bounds.

The cell-specific metabolic rate, m, is given in femtograms of carbon per cell per day by:


(1) $$m=\frac{\left(S_{0}+S_{i}\right)-S_{f}-\alpha_{D}\left(N_{f}-N_{0}\right)}{\int_{0}^{f}N\left(t\right)dt}$$

where $\alpha_{D}$ denotes the quantity of organic carbon content per cell in femtograms of carbon per cell; S , the concentration of TOC in femtograms of carbon per milliliter; N , the cell density in number of cells per milliliter, and 0 and f , the start and end times, respectively, in days. $S_{i}$ is any organic carbon added at time i . Sympy v1.11.1 was used to calculate the integral (Meurer et al., 2017). We took $N_{f}$ to be the observed cell density in the cryopeg brine of reference for each scenario (Table 1). We took $N_{0}$ to be 10^5^ cells mL^−1^, the order of magnitude of cell densities observed in coastal sea ice (Cooper et al., 2019). The use of a coastal sea ice value is based on physical similarities between the two environments, and the potential that coastal sea ice of 40,000 years ago, being a frozen, brine-containing surface environment, supported a microbiome resembling that of cryopeg brine when first formed.

### 3.5. Extracellular enzyme activity

Measurements of EEA in cryopeg brines are available (Showalter, 2020), but we sought to calculate EEA rate bounds to understand whether EEA might hint at the age and energetic requirements of the microbial community. These bounds were calculated similarly to those for the cell-specific metabolic rate, using Equation 2:


(2) $$\gamma_{\mathrm{cell}}=\frac{\left(P_{0}+P_{i}\right)-P_{f}}{\int_{0}^{f}N\left(t\right)dt}$$

where cell-specific EEA rate, $\gamma_{\mathrm{cell}}$ , is given in femtograms of carbon per cell per day^,^ and P denotes quantity of POC. We thus considered the difference in POC quantity between the brine and its surroundings, accounting for any additional POC input to the brine, with the resulting quantity divided by cell density over time. The bounding growth trajectories were identical to those used to estimate the cell-specific metabolic rate. Using Equation 2, we could also predict the timespan of the system by replacing $\gamma_{cell}$ with measured EEA rate and solving for t . We solved for the timespan using the growth case with the minimum calculated growth rate starting at 10^5^ cells mL^−1^.

### 3.6. Model description

**Figure 3:**
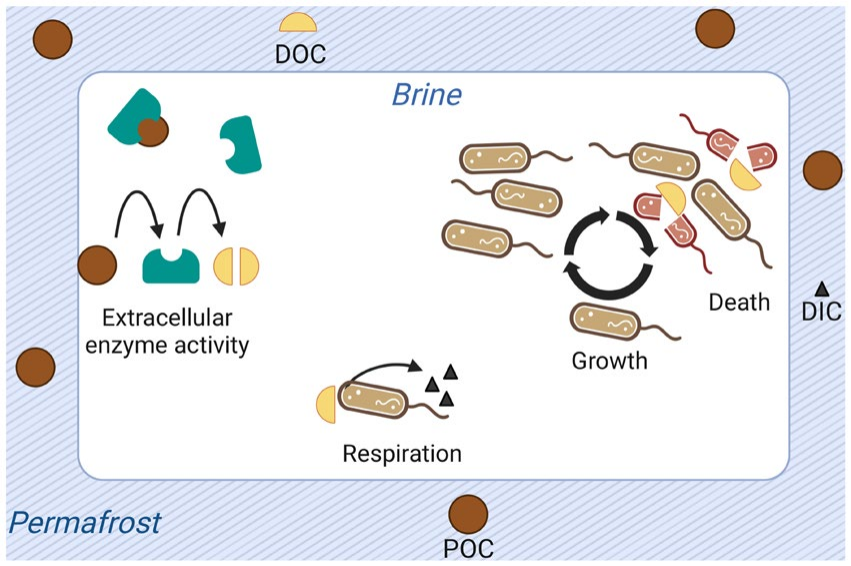
Graphical representation of processes and quantities modeled for permafrost-enclosed cryopeg brine. Particulate organic carbon (POC) is shown as dark brown circles; dissolved organic carbon (DOC), as yellow semi-circles; and dissolved inorganic carbon (DIC), as black triangles. Extracellular enzymes (teal-colored shapes) produced and released by bacteria hydrolyze POC to DOC. Bacteria take up DOC, respiring it to dissolved DIC or assimilating it into biomass to grow and reproduce. They release DOC back to the brine upon death, attributed explicitly to starvation in the model.

To determine the plausibility of the calculated cell-specific metabolic rate value, and to understand the energetic requirements of the system, we developed a model of the organic carbon cycle in Utqiaġvik cryopeg brines. In this section we offer a non-mathematical description and schematic representation (Figure 3) of the model. The central assumptions are presented in the next section.

The model keeps track of four quantities: POC, DOC, DIC, and cell abundance. Five processes structure these quantities: respiration of DOC to DIC by bacteria, cell growth, cell death, hydrolysis of POC to DOC by extracellular enzymes, and organic carbon additions to the system.

Here the rate of respiration, as well as all other utilizations of organic carbon (e.g., cell growth, production and release of extracellular compounds), is encompassed in the cell-specific metabolic rate. Each unit of organic carbon respired is removed from the DOC pool and added to the DIC pool. The quantity of DIC in the model runs presented here are modified solely by this cellular respiration of DOC. Cells grow according to an equation relating their maximum growth rate to substrate concentration (Monod, 1949). As a cell grows, the quantity of DOC it contains is removed from the DOC pool. Conversely, cells release the DOC they contain upon death. Cell death is explicitly considered to occur when the quantity of available DOC is less than the cell-specific metabolic rate and thus is referred to as starvation death. Other death processes are abstracted by net growth rate. Extracellularly, the enzymatic conversion of POC to DOC contributes to the DOC pool.

As used in the CBIW scenario, the model allows for the addition of organic carbon to the system, beyond that available at time zero. A model feature not used here would allow consideration of a punctuated or a constant input of DIC through processes other than respiration, such as carbonate dissolution. Possible chemoautotrophy to remove DIC and generate new biomass (Rapp et al., 2021) would require modifications that are being considered for a future modeling effort.

### 3.7. Model assumptions and limitations

Due to the sparse data available on cryopeg brines, reconstructing their microbial history requires assumptions to obtain the first-order approximation of their energetics. These assumptions inherently introduce limitations to our results. We address the central assumptions here to contextualize our results.

A key assumption in our analyses is that the cryopeg brines started with an amount of organic carbon equivalent to the quantity observed in their contemporary surroundings. This assumption is required, as obtaining precise information on a cryopeg system at the time of its formation 40,000 years ago is not possible. The assumption is not unreasonable given the hydrological isolation of the brines and the temperatures that have kept their surroundings frozen throughout their lifetimes (Iwahana et al., 2021; Osman et al., 2021).

Our model does not account for diffusion of material throughout the brine, which neglects the likelihood of environmental niches (Rapp et al., 2021). Without *in situ* microscale observations of these remote systems, we cannot parameterize niches or differentiate the energetic requirements of inhabiting them. For the purposes of conducting an overarching energetic analysis of the system, the lack of diffusivity may not be a critical limitation. The analysis is simply spread uniformly across the environment and the microbes.

We assumed that every cell in the system grows at the same rate. Of course, different subpopulations of cells express different phenotypes and levels of activity, including dormancy, at different times in their life histories. This assumption likely leads to an overestimation of the community growth rate and, in turn, an underestimation of the cell-specific metabolic rate. However, as will be seen in our results, the cell-specific metabolic rate compares reasonably well to existing estimations.

We also assumed that every cell in the system has the same carbon content throughout its lifetime. Of course, bacterial communities exhibit a range of cell sizes, with size and potentially content changing as a function of growth conditions, growth phase, starvation conditions, and dormancy. Until distribution data for cryopeg brines are obtained, the use of a uniform distribution of cell size and carbon content in our analyses leaves some uncertainty to our results. As will be seen, our model simulations are not overly sensitive to this parameter.

We have attributed cell death to starvation, but other processes can lead to cell death in these cryopeg brines. In particular, cell lysis following viral infection and “bacterial warfare” may contribute significantly (Rapp et al., 2021). Use of a net community growth rate includes such death processes implicitly. Our use of growth rates determined in cultures as net community growth rates likely represents an overestimation of the net rate. As a future research direction, this model could be modified to account explicitly for death mechanisms other than starvation.

We assumed that microbial community kinetics could be represented by those of the dominant bacterium. Many bacterial species exist within the cryopeg brine, each with presumably distinct kinetics. However, overall diversity was low and the dominant species was strongly dominant in each scenario considered, accounting for half or more of the community. Making this assumption allowed a first-order approximation despite the unknown complexities of community kinetics.

We assumed that all POC is inaccessible to the brine community until hydrolyzed enzymatically to DOC, and that all DOC is accessible and labile. An absence of data on the chemical composition and lability of either of these pools of organic carbon limits our analysis, as does the assumption that EEA rate is constant. Bacteria regulate their production of extracellular enzymes in response to environmental substrates, but we lack data to model this kinetic or enzyme lifetime. These assumptions could lead to biased results in EEA rate calculations and final carbon quantities (Supplementary Figure S1).

Finally, we assumed that organic compounds are the only limiting source of carbon, nutrients and energy for cell respiration and growth. Sources of inorganic nutrients are plentiful in cryopeg brines (Cooper et al., 2019), but are not included explicitly in our model. While data on the existence of other sources of chemical energy in the system are limited, the levels of POC and DOC in the brines indicate this assumption to be reasonable. The microbial communities in the brines examined were dominated overwhelmingly by organoheterotrophs, further supporting the assumption.

### 3.8. Model equations

The model was solved using the DifferentialEquations.jl package v7.6.0 (Rackauckas and Nie, 2016) using a Rosenbrock23 solver (Shampine and Reichelt, 1997) set up to solve an initial-value problem.

The growth term, G, is solved with a straightforward Monod equation (Monod, 1949) that relates the maximum net growth rate, $\mu_{\max},$ to substrate concentration, D, using a half-velocity term, $K_{D}$ , where the substrate is DOC (Equation 3):


(3) $$G=\mu_{\max}*\frac{D}{K_{D}+D}*N*\left(1-\frac{N}{N_{\max}}\right)$$

A logistic growth term has been added to cap the growth as the cell density, N, approaches carrying capacity, $N_{\max}$ . If cell density declined to zero at the time of a carbon addition (e.g., in the CBIW scenario), we set N=1 to simulate a viable cell able to respond to the addition.

The death term, Δ , corresponds to deaths by starvation (Equation 4):


(4) $$\Delta =\frac{\max\left(mN-D,0\right)}{m}$$

This term accounts for the assumption that cells will lyse if they do not have enough substrate to maintain their integrity, i.e., cannot satisfy their metabolic need ( m ). Other death-inducing processes such as viral infection or bacterial warfare are included implicitly in net growth rate (Equation 3). The net change in cell density is the difference between growth and death by starvation (Equation 5):


(5) $$\frac{dN}{dt}=G–\Delta$$

While the cell-specific EEA rate remains constant throughout our simulations, the absolute EEA rate, γ , must be lower or equal to the available quantity of substrate, P . To satisfy this constraint, we used a minimum function (Equation 6):


(6) $$\gamma =\min\left(\gamma_{cell}*N,P\right)$$

where P is the quantity of POC, which is given by the sum of two terms. The first term, $P_{in,}$ represents any addition of POC into the system. The second term is the absolute EEA rate, γ, subtracted to remove the quantity of POC hydrolyzed to DOC.


(7) $$\frac{dP}{dt}=P_{in}-\gamma$$

The quantity of DOC in the system is the sum of five terms (Equation 8):


(8) $$\frac{dD}{dt}=D_{in}+\alpha_{D}\left(\Delta -G\right)-m\left(N-\Delta \right)+\gamma +\min\left(I_{xr},I\right)$$

The first term is any addition of organic carbon into the system, $D_{in}$ , which allows the simulation of single or repeated carbon additions from the surrounding environment into the cryopeg brine. The second term is DOC sequestered or released by biomass, equal to the change in cell abundance multiplied by the quantity of organic carbon per cell, $\alpha_{D}$ . The third term accounts for the metabolism of the remaining population. The fourth term, γ, is the quantity of POC converted to DOC by EEA. The final term simulates autotrophy in the system by taking a DIC fixation rate, $I_{xr},$ which removes carbon from the DIC pool and adds to the DOC pool. This rate cannot be smaller than the quantity of DIC. In this work, we have set this term to zero, for lack of measurements of $I_{xr}\;$ in cryopeg brines and expecting the rate to be minor based on metagenomic information (Rapp et al., 2021). If such rates become available for future simulations, the model would need modification to accommodate the flow of DIC into new cell biomass instead of directly into DOC (Section 3.7).

The equation for change in DIC is constructed similarly, though the EEA term is absent as it need not be considered (Equation 9):


(9) $$\frac{dI}{dt}=I_{in}+\alpha_{I}\left(\Delta -G\right)+m\left(N-\Delta \right)-\min\left(I_{xr},I\right)$$

In this equation, $\alpha_{I}$ is the quantity of DIC per cell. While $\alpha_{I}$ and $I_{xr}\;$ are set to zero here, they could be used in a future study to model autotrophy in this system.

### 3.9. Model inputs and simulations

In addition to the variables defined for each cryopeg brine scenario (Table 1), a set of constants were input to the model (Table 2). For each constant, we used the most accurate estimate we could find. In some cases, the chosen value was less relevant to our unique environment than we had hoped. However, given that we are striving for order of magnitude estimations, we expect these to be adequate, especially when considering the results of our sensitivity analysis (Sections 3.10 and 4.4).

**Table 2:** Constants used to model cryopeg brine scenarios.

Constant	Symbol (unit)	Value	Reference
Carrying capacity	$N_{\max}$ (cells mL^−1^)	10^9^	Assumed, based on Schmidt et al. (1998)
Monod half-velocity constant	$K_{D}$ (fg C mL^−1^)	8.82 × 10^5^	Estimated from Rowe and Deming (1985) and Yager and Deming (1999)
Cell density at $t_{0}$	$N_{0}$ (cells mL^−1^)	10^5^	Assumed, based on density in coastal sea ice (Cooper et al., 2019)
Extracellular enzyme activity rate	$\gamma_{\max}$ (fg C cell^−1^ day^−1^)	1.22 × 10^−2^	Measured in CBIW (Showalter, 2020)
Simulation timespan	$t_{f}$ (years)^a^	40,000	Measured (Iwahana et al., 2021)

^a^Converted to days for all calculations.

$K_{D}$ was derived by taking an average of the values for amino acid uptake rates measured at −1°C in Arctic seawater by Yager and Deming (1999), then taking the dissolved combined amino acids to be 41% carbon (as in Rowe and Deming, 1985). No adjustment was made for temperature, as the original data, determined across a range of temperatures, showed no conventional Q10 effect (Yager and Deming, 1999).

We ran simulations to obtain growth trajectories (changes in cell density over time) for each of the three cryopeg scenarios under different boundary conditions. Each simulation represents one of 8 unique combinations of the lower or upper bound of three variables: m , $\mu_{\max}$ , and $\gamma_{cell}$ . Simulations thus address minimum and maximum growth rate, lower and upper bound cell-specific metabolic rate, and calculated and measured extracellular enzyme activity. They also track POC and DOC during the 40,000-year time span of the resulting growth trajectories.

### 3.10. Sensitivity analysis

To understand how the accuracy of our estimates affect model results, we conducted a sensitivity analysis of model parameters. Using GlobalSensitivity.jl package v2.1.2 (Ma et al., 2021) we executed a global sensitivity analysis using a variation of the Sobol variance decomposition method and estimator (Sobol, 2001; Sobol et al., 2007). The analysis was allowed to converge to obtain a satisfactory confidence interval at a confidence level of at least 95%.

Briefly, this variance decomposition method varies each parameter within given bounds and measures the corresponding variance of the output. The resulting first-order Sobol index of a parameter is a measure of how much variance in the output can be attributed to that parameter. The total-effect Sobol index encompasses interactions between the variance of that parameter and the others in the analysis. The bounds passed for each parameter aim to encompass the range of microbiologically plausible values; these can be found in Table 3.

**Table 3:** Parameter bounds of the sensitivity analysis.

Parameter (units)	Lower bound	Upper bound
Growth rate (day^−1^)	1 × 10^−6^	1 × 10^2^
Cell-specific metabolic rate (fg C cell^−1^ day^−1^)	1 × 10^−5^	5 × 10^2^
Cell carbon content (fg C cell^−1^)	1 × 10^2^	5 × 10^2^
EEA rate (fg C cell^−1^ day^−1^)	0	1 × 10^2^
Monod half-velocity constant (fg C)	1 × 10^3^	1 ×10^8^
Carrying capacity (cells mL^−1^)	1 × 10^8^	1 × 10^9^
Starting cell density (cells mL^−1^)	1	1 × 10^8^

The sensitivity analysis was executed on the CB1 scenario, excluding environmental parameters. This choice was made to understand the influence of bacterial parameters, such as carbon content per cell and growth rate, on our results. Furthermore, these constitute the few parameters that future lab work may attempt to quantify, whereas more accurate environmental data is elusive.

## 4. Results

Here we provide the results of the different measurements made to fill gaps and complement the datasets available in the literature, and thus enable our model simulations. These measurements include sediment carbon and nitrogen content of cryopeg and regional sediments and brine DIC for one borehole sample. Estimates of cell-specific metabolic rate and extracellular enzyme rate are shown for the three different scenarios examined. Finally, a sensitivity analysis of model parameters and the model predictions are presented.

### 4.1. Sediment carbon and nitrogen measurements

To improve the accuracy of our model, we measured the quantity of organic carbon in sediment permafrost previously sampled at two locations in the BEO. The average value for POC was 0.0232 ± 0.0006 μg C μg sediment^−1^ at a depth of 367–383 cm (*n* = 4). The porewater salt concentration was 6‰. The concentration of DOC in this porewater was 51.2 μg C mL^−1^. The nitrogen content of the sediment was 0.0016 ± 0.00005 μg N μg sediment^−1^, for a molar C:N ratio of 15.8 mol C mol N^−1^.

We also measured the concentration of organic carbon in sediment surrounding the CB4 cryopeg brine. For POC, the average value in this sediment layer was 0.0136 ± 0.0012 μg C μg sediment^−1^ (*n* = 3). The porewater salt concentration ranged between 22‰ and 30‰. The concentration of DOC in this porewater at 187-cm depth was 1,286 μg C mL^−1^. The nitrogen content of the sediment was 0.0010 ± 0.00015 μg N μg sediment^−1^. CB4 sediment thus had a C:N ratio of 15.8 mol C mol N^−1^.

### 4.2. Brine dissolved inorganic carbon

The dissolved inorganic carbon content of CBIW brine (in 2017) was measured as 6.93 × 10^10^ fg C mL^−1^. The dissolved inorganic carbon content of frozen sediment from CBIW (in 2018) was 4.9 × 10^10^ fg C mL^−1^. We do not have DIC measurements for other brines.

### 4.3. Estimates of cell-specific metabolic rate

The lower and upper bounds of the cell-specific metabolic rate that we estimated for each of the three cryopeg brine scenarios ranged from 0.008 to 0.743 fg C cell^−1^ day^−1^ (Table 4). As expected, due to the input of organic carbon, the lowest of these rates was obtained for the CBIW scenario. The calculated minimum growth rate ranged between 2.77 × 10^−7^ and 4.95 × 10^−7^ day^−1^. These extremely low growth rates, which correspond to doubling times on the order of 10^3^ years, are due to the significant age of the system and rely upon our assumption of a starting cell population of 10^5^ cells mL^−1^.

**Table 4:** Cell-specific metabolic rate bounds and calculated maximum doubling time and minimum growth rate for each cryopeg brine scenario.

Parameter (units)	CB1 scenario	CB4 scenario	CBIW scenario
Cell-specific metabolic rate (fg C cell^−1^ day^−1^)	0.181–0.743	0.099–0.474	0.008–0.057
Maximum doubling time (years)	6,860	5,850	3,870
Minimum growth rate (day^−1^)	2.77 × 10^−7^	3.24 × 10^−7^	4.95 × 10^−7^

### 4.4. Extracellular enzyme activity rate estimates and predicted timespan

We calculated the EEA rate required to hydrolyze the difference between surrounding sediment POC and brine POC. In the case of the CB1 scenario, the bounds of estimated EEA rates were 0.195 and 0.802 fg C cell^−1^ day^−1^. At the measured EEA rate of 0.012 fg C cell^−1^ day^−1^, the time required to hydrolyze the POC difference in this scenario would have been 81,200 years. In the CB4 scenario, the estimated bounds of EEA rates were 0.101 and 0.483 fg C cell^−1^ day^−1^, with a predicted timespan of 71,000 years. Finally, in the CBIW scenario (that received organic input), the estimated bounds of EEA rates were 0.806 and 0.584, with a predicted timespan of 48,700 years, much closer to the observed age of the cryopeg system. In all cases, this independent estimation of the system’s timespan is near to or less than double the measured timespan, well within the goal of obtaining order of magnitude estimates.

### 4.5. Model sensitivity analysis

**Figure 4:**
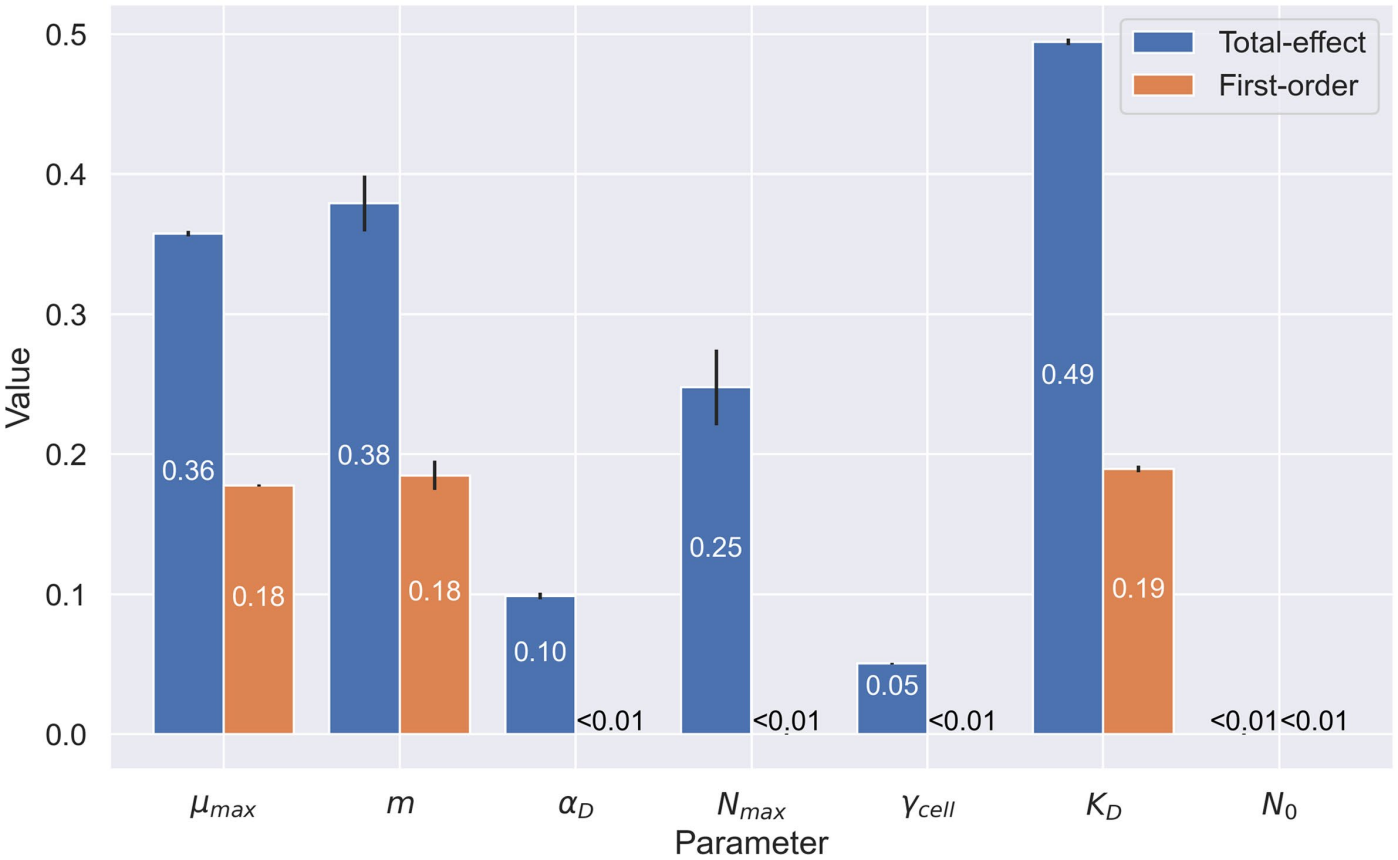
Sensitivity analysis of microbial parameters used in the organic carbon model. Numerical values represent the first-order (in orange) and total-effect (in blue) Sobol indices of the selected parameters: maximum growth rate ( $\mu_{\max}$ ), cell-specific metabolic rate ( m ), cell carbon content ( $\alpha_{D}$ ), carrying capacity ( $N_{\max}$ ), cell specific extracellular enzyme activity ( $\gamma_{cell}$ ), half-velocity constant for carbon uptake ( $K_{D}$ ), and starting cell density ( $N_{0}$ ). First-order indices show the sensitivity of the model when varying only the parameter in question. Total-effect indices show the sensitivity of the model when varying the selected parameter in conjunction with the other parameters selected in this analysis. Values shown are rounded at 10^−2^.

A sensitivity analysis of model parameters revealed the importance of the three parameters at the heart of the Monod growth term (Figure 4): growth rate, cell-specific metabolic rate, and half-velocity constant. These are the only parameters with notable first-order indices. They have a measurable direct impact on model output, whereas cell carbon content and initial cell density do not. The total-effect indices offer more nuance. These indices reflect the added importance of carrying capacity. As a whole, this sensitivity analysis offers insight into which parameters account for much of the variability in the model results.

### 4.6. Model predictions

**Figure 5:**
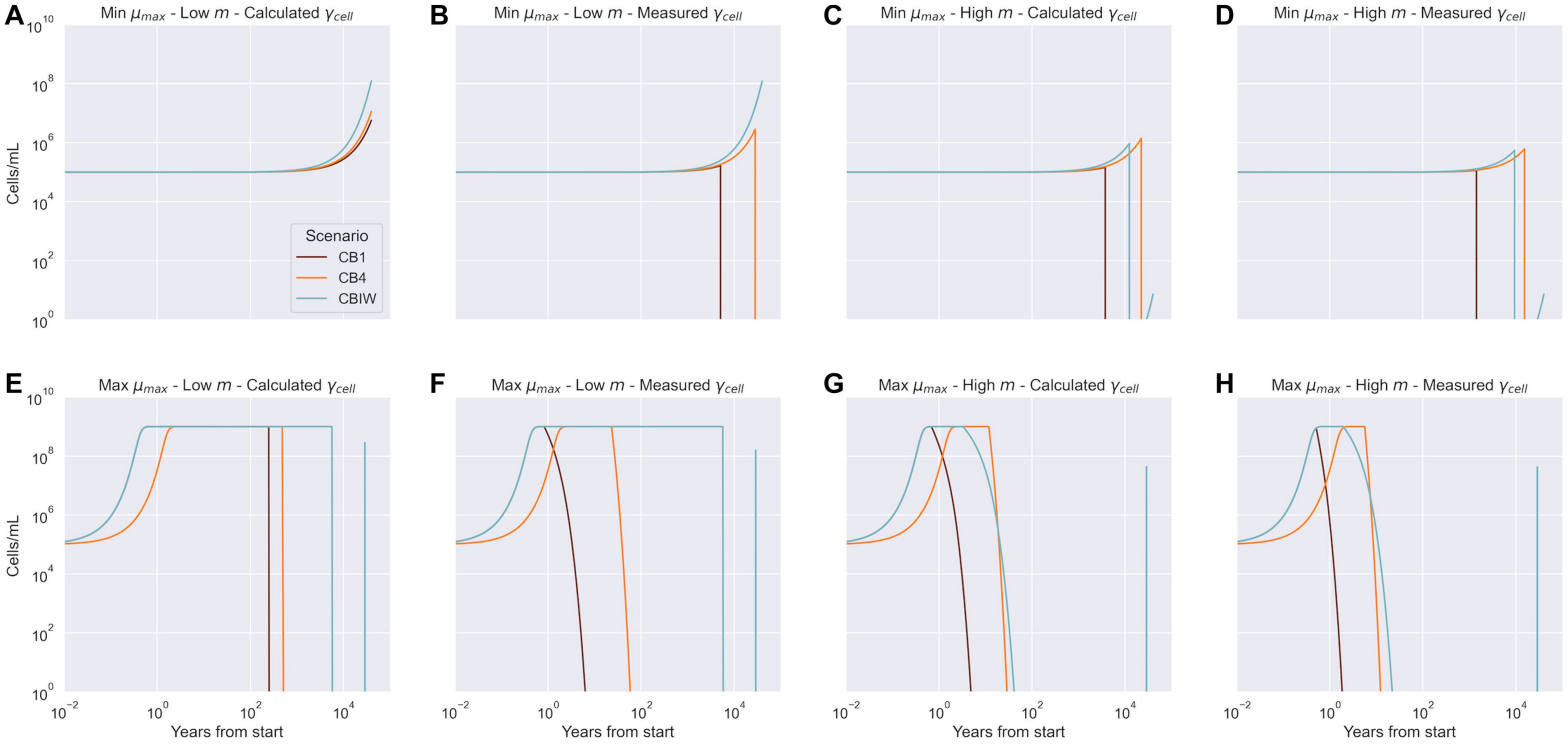
Model predictions of cell density over the lifetime of the system for each cryopeg brine scenario. Measured cell densities at 40,000 years are recreated by the model only under conditions shown in panel **A**: lower bound net growth rate ( $\mu_{\max}$ ), lower bound cell-specific metabolic rate ( m) , and the calculated cell-specific extracellular enzyme activity ( $\gamma_{cell}$ ); and for CBIW in panel **B**: lower bound net growth rate ( $\mu_{\max}$ ), lower bound cell-specific metabolic rate ( m) , and the measured cell-specific extracellular enzyme activity ( $\gamma_{cell}$ ). Panels **C** through **H** depict cases where the predicted end cell density did not match the observed cell density, regardless of the combination of bounds applied.

In two of the eight sets of conditions used for model simulations, our model fully or partially succeeds in explaining the cell densities observed at 40,000 years. With conditions of minimum growth rate paired with low cell-specific metabolic rate and calculated EEA, all three cryopeg brine scenarios reach their observed cell densities (Figure 5A). With measured EEA (Figure 5B), the observed cell density is reached only for the CBIW scenario; CB1 and CB4 scenarios fall short of their densities (Figure 5B). The CBIW scenario succeeds in reaching the observed cell density because it includes an addition of DOC, while the other two depend upon the hydrolysis of existing POC to generate DOC for bacterial growth. Insufficient POC is hydrolyzed toward the end of the trajectory (Supplementary Figure S1) to support these other two communities.

By Equation 1, minimum growth rate paired with the upper bound cell-specific metabolic rate should yield the observed cell densities. However, this is not the case. Common to all the minimum growth rate simulations (Figures 5A–D) is a long plateau for the first few thousand years. In those simulations where minimum growth rate and upper bound cell-specific metabolic rate are paired, the populations subsequently grow weakly before declining (Figures 5C,D). Given that abundant POC remains in the system in all cases (Supplementary Figure S1), these results are explained by an EEA rate inferior to the cell-specific metabolic rate requirements. The CBIW population does start to recover due to addition of DOC at 29,000 years, but the growth rate is too low to allow recovery to the observed cell densities before the simulation ends (Figures 5C,D).

When the maximum growth rate is used, the microbial populations rapidly reach the system carrying capacity (Figures 5E–H). Once they consume all available DOC, the populations decline. The time elapsed before the decline is governed by the demand for DOC driven by the cell-specific metabolic rate. Because CBIW sees a punctual addition of DOC at 29,000 years, the high growth rate allows the population to recover for a short time before declining again (Figure 5 panels E-H). However, not enough carbon has been added to sustain the population to the end of this simulation.

## 5. Discussion

Our overall approach can be considered a bulk energetic analysis. We have made the fundamental assumption that the total energy use of the system corresponds to the difference in organic carbon between the permafrost and the cryopeg brine, assuming these started with equal amounts. This approach mitigates the lack of a secondary metabolic state (i.e., dormancy) in our model. In this way, our cell-specific metabolic rate calculations provide bounds on the average energy requirement of a bacterium in this setting.

Multiple analyses increase our confidence in the mentioned assumption and our model predictions. Using the measured rate of EEA and following our assumption on total energy use in this system, we calculated an expected timespan of the system equal or less than double the measured timespan. We also used the measured C:N ratio of CB4 sediment and the previously measured quantity of ammonia in the brine from Cooper et al. (2019) to estimate the amount of organic carbon consumed. We obtained a value of 8.59 × 10^11^ fg C mL^−1^, two orders of magnitude below the quantity produced by our assumption. This calculation is expected to produce a lower bound quantity given that it does not consider nitrogen cycling or the concentration of other nitrogen species in the brine. Thus, this quantity does not contradict our assumption or prediction. Similarly, the DIC value measured is three orders of magnitude below our expectations based on DOC consumed in CBIW. This analysis indicates that we may be overestimating DIC in the model by not accounting for the complexities of the inorganic carbon system in a subzero brine (Marion et al., 1999), supported by the measured DIC in surrounding frozen sediment. Together these results confirm the plausibility of our assumptions, while providing crucial context for our results. Future work could focus on modeling the DIC sinks of these brines in order to increase the fidelity of this model and understand the potential role for autotrophy in this extreme microbial community.

To understand the biological plausibility of our metabolic rate estimates we sought to compare them to existing measurements and estimates of metabolic rates in other remote and energy-limited environments. Assuming an energetic yield of 30 kJ mol C^−1^ our cell-specific metabolic rate values range on the order of 10^−17^ to 10^−19^ W cell^−1^. This range overlaps at the high end of the range for estimated energy turnover from cold anoxic subsurface marine sediments, the closest analog we can find, on the order of 10^−19^ W cell^−1^ and 10^−20^ W cell^−1^ (Hoehler and Jørgensen, 2013; Lever et al., 2015). For deep crustal fluids, another energy-limited but open and oxygenated environment, sample incubations amended with ^13^C-substrates at 4°C yielded potential anabolic rates that range from 10^−3^ to 30 fg C cell^−1^ day^−1^ (Trembath-Reichert et al., 2021). Our metabolic rates, which represent net anabolic and catabolic activities at −6°C, fall at the low end of this range (order of 10^−3^ to 10^−1^ fg C cell^−1^ day^−1^; Table 4), as expected given the significant differences between these environments.

Thus, the cell-specific metabolic rate values we obtained, being framed by other existing rates, are biologically plausible. Our calculation of the cell-specific metabolic rate implicitly includes every cellular process but growth, including membrane, protein and other adaptations to low temperature and high salinity. The relatively high cell-specific metabolic rate of bacteria in the cryopeg brine system, compared to deep subsurface marine sediments, can be accounted for at least partly by the production of extracellular compounds. Moreover, cell carbon mass in these environments may be larger than cells in deep sediments, which could also account for some of the difference in cell-specific metabolic rate between cryopeg brines and cold (unfrozen) marine sediments.

Extracellular enzyme activity has been documented in cryopeg brines (Showalter, 2020). Producing extracellular enzymes is a costly endeavor, with the price reflected in higher cell-specific metabolic rate requirements (Vetter et al., 1998; Lever et al., 2015). Extracellular polysaccharides are also present in high concentrations in cryopeg brines (Cooper et al., 2019). These polysaccharides take on many critical functions, offering cell protection against the effects of both subzero temperatures and hypersaline conditions (Krembs et al., 2002, 2011; Carillo et al., 2015; Deming and Young, 2017). While extracellular polysaccharides appear to be fundamental in allowing a cryopeg microbial community to exist, their chemical nature and high concentration in cryopeg brines implies a significant energetic cost, again implicitly built into our cell-specific metabolic rate calculation.

We calculated the minimum growth rate of bacteria in this system, making an important assumption on the starting cell concentration of these brines. While we have made an informed assumption in using cell density in sea ice, the value remains at best an educated guess. We have no data on the actual starting cell concentration of these cryopeg brines. Nevertheless, our sensitivity analysis suggests little influence of this parameter on model output. Assuming a starting cell concentration of 10^5^ cells mL^−1^, we obtain maximum doubling times on the order of 10^3^ years. This result is microbiologically feasible, based on similarly long doubling times (20–2,500 years) calculated for other, related energy-limited environments, particularly marine and deep subsurface sediments (Hoehler and Jørgensen, 2013; Lever et al., 2015; Jørgensen and Marshall, 2016), which are thermally more growth-permissive environments than subzero cryopeg brines. As we discuss below, very slow doubling times are not necessary in all cases to explain our observations.

The sensitivity analysis reflects the importance of the parameters we chose to manipulate in our simulations. Growth rate, cell-specific metabolic rate, and Monod half-velocity constant each have high first-order and total-effect Sobol indices. These high indices reflect an important interplay between the parameters in determining the outcome of the model. The EEA rate also presents a high total-effect index in its role of determining the quantity of DOC in the system. Surprisingly, EEA rate does not exhibit a high first-order index, despite other data suggesting this variable is key to cryopeg brine energetics. In fact, EEA does have a high Sobol index when only considering the end concentration of POC in the system (not shown).

In all but two (of 8) sets of conditions for simulations, the model fails to reconstruct a cell growth trajectory that yields the observed cell densities. In these cases, cell density collapses to zero due to the lack of available DOC to meet community energetic requirements. However, in most cases, plenty of POC, a potential source of energy, remains (Supplementary Figure S1). The extinction of available DOC in these simulations (Supplementary Figure S1) is caused by the cell-specific metabolic rate being higher than the cell-specific EEA rate. In other words, individual cells are consuming DOC faster than their enzymes can convert POC to DOC.

While the half-velocity constant presents high Sobol indices, this result must be nuanced. First, the chosen value is orders of magnitude lower that the quantity of DOC present in the system. Thus, the impact of this term at the beginning of the simulation is low, and growth proceeds unimpeded. A higher value might slow growth, but the timespan here is long enough that the effect would be negligible. When the quantity of DOC in the system is closer to or below the value chosen, the community is typically on a trajectory toward extinction in the model. Hence, the half-velocity constant, while important because it modulates growth, does not alone explain why the model is limited in explaining our observations.

Simulations based on the CBIW scenario provide some insight on model success versus failure in predicting the observations. In this scenario, the lower bound cell-specific metabolic rate is lower than the extracellular enzyme activity rate used, whether the calculated or measured value (Table 4, Section 4.3). Thus, with abundant DOC available, the model can reproduce the observed cell density using the minimum growth rate (Figures 5A,B). Observed cell densities are also obtained for the CB1 and CB4 scenarios if the calculated EEA rate is used (Figure 5A). In contrast, using the maximum growth rate in conjunction with the calculated EEA rate leads to the depletion of the POC pool, unless carrying capacity is reduced by one order of magnitude (not shown).

Clearly, the EEA rate has the potential to make bioavailable a significant energy source in the system: POC. We note that the observed cell-specific rate of EEA used in the simulations is the average of activities measured on only three substrates in CBIW brine. This rate is kept constant throughout every simulation. This simplification does not accurately reflect the number and specificity of enzymes produced in a cryopeg brine, their complex kinetics, the regulation of their production, or their lifetimes under subzero brine conditions. The cell-specific EEA rate surely fluctuated over the lifespan of the brine as a function of cell density, DOC concentration, and extracellular enzyme turnover time. We were thus led to calculate the range of EEA rates that would allow for enough POC to be hydrolyzed to sustain the microbial community. For the CB1 and CB4 scenarios, these calculated rates are an order of magnitude higher than the measured rate used. Conversely, we calculated the amount of time needed for the chosen EEA rate to hydrolyze the required amount of POC. The results point to a longer lifespan for the cryopeg brines than the measured 40,000 years (by carbon dating), as high as 81,200 years in one case. This discrepancy, based on the chosen EEA rates being too low, provides one explanation as to why our model fails in many cases to explain the observed cell densities. A lack of data to account for cell size differences between *in vitro* and *in situ* measurements may represent another. Future research on extracellular enzyme production and kinetics at subzero temperatures and high salinities, coupled with cell-size measurements, could allow for an improved understanding of bacterial energetics in cryopeg brines.

Despite being detected (Colangelo-Lillis et al., 2016; Cooper et al., 2019), hydrogen sulfide is not considered here as a potential source of chemical energy in these cryopeg brines. Most members of the bacterial communities as revealed by metagenomics are not known to utilize it (Rapp et al., 2021), bacterial kinetics for those that do are not available under *in-situ* conditions, and hydrogen sulfide concentrations are not known. However, the presence of sulfur-oxidizing and sulfur-reducing bacteria was recorded in these brines (Cooper et al., 2019). Therefore, a large enough endogenous source of hydrogen sulfide in the cryopeg brines could change the energetic balance of these communities. We also note that sulfur concentration may be impacted by abiotic processes such as mirabilite precipitation (Marion et al., 1999). Armed with the relevant kinetics, a future study could establish an upper bound on the possible energetic contribution of hydrogen sulfide to this system. A more detailed analysis could account for metabolically different bacterial populations and different metabolic states.

## 6. Conclusion

Here we have produced a first estimation of cell-specific metabolic rate in cryopeg brines, ancient, geologically isolated, subzero hypersaline liquids in Arctic permafrost. Comparing our estimates to the few other estimates available on natural microbial systems is difficult, given the use of different approaches and reporting units. Our estimates suggest that cell-specific metabolic rate in a cryopeg system is much higher than in subsurface marine sediments (Hoehler and Jørgensen, 2013), the closest parallel we can find. Although both are sediment-based systems, the differences between them are marked: subsurface marine sediments are neither subzero nor hypersaline and they lack the energy resources that surround a cryopeg system. However, our estimates of cell-specific metabolic rate may be high primarily because we define cell-specific metabolic rate to include every cellular process but growth. The energetically costly production of extracellular enzymes and extracellular polysaccharides may best explain our result of high cell-specific metabolic rate.

To further understand the energetics of the cryopeg system, we developed a model of a simplified organic carbon cycle in subzero cryopeg brines. The results of a selective sensitivity analysis of this model suggest that growth rate, cell-specific metabolic rate and EEA rate are key parameters in determining the fate of the microbial community. Running simulations representative of different energetic bounds for each cryopeg brine improved our understanding of the history of a cryopeg microbial community. In most cases, the energetic requirement is too high and the microbial community collapses. A higher EEA rate would allow the community to take advantage of the energy locked within the high amounts of POC in the system. Where we modeled a punctual addition of DOC into the system, the population was either too slow growing to achieve the observed cell density, or the quantity of DOC was insufficient to sustain it. In cases where the lower bound cell-specific metabolic rate was inferior to the EEA rate, the model was successful in reproducing the observed cell densities after 40,000 years. In calculating the required EEA rate to satisfy the energetic requirements in each of the cryopeg brine scenarios considered, we concluded that the 40,000-year timespan could be reconciled with the measured EEA rate if the energetic requirements of the bacterial community are low enough. The calculated EEA based on our assumption on starting DOC and POC conditions yields an expected system timespan within a factor of two of the measured timespans. This result increases our confidence in the assumptions underlying our energetic analyses.

Although our model unavoidably relies on numerous assumptions, it has produced testable hypotheses for the continued study of cryopeg brines. It also leads us to conclude that the microbial densities observed today in cryopeg brines could well have been reached in energetic isolation over an estimated system lifespan of 40,000 years. In general, the success of these communities would have required a lower growth rate than that observed under *in-situ* conditions in the lab and higher average rate of EEA. The calculated cell-specific metabolic rate of bacteria in these systems can be met by the assumed available quantity of POC and DOC in the system and appears to be biologically plausible, particularly for bacteria functioning under the extreme conditions of subzero temperature and hypersalinity. Finally, this model could be tuned in its parameters to describe theoretical astrobiologically relevant environments within the icy crusts of Europa or Enceladus.

## Data availability statement

The original contributions presented in the study are included in the article/Supplementary material, further inquiries can be directed to the corresponding author. The code to reproduce the analyses in this study can be found in the GitHub repository of this project at https://github.com/Ge0rges/Cryopeg-Carbon-Model.

## Author contributions

GK developed the carbon model, estimated the cell-specific metabolic rate values, performed all calculations, model runs, and lab work, and drafted the manuscript. TH provided the methodological ideas required to estimate cell-specific metabolic rate, crucial feedback on the model, including the suggestion to conduct a sensitivity analysis, and critical revisions to the manuscript. GI provided organic and inorganic carbon data, expansion factors for ice, interpretation of dating measurements, and edits to the manuscript. JD supervised all work, advised GK, administered the grant, provided lab facilities, and revised and edited the manuscript. All authors approved this submission.

## Funding

This work was initially supported by the Gordon and Betty Moore Foundation (grant number GBMF5488). GK also received support from a private sponsorship and the Karl M. Banse professorship to JD.

## Conflict of interest

The authors declare that the research was conducted in the absence of any commercial or financial relationships that could be construed as a potential conflict of interest.

## Publisher’s note

All claims expressed in this article are solely those of the authors and do not necessarily represent those of their affiliated organizations, or those of the publisher, the editors and the reviewers. Any product that may be evaluated in this article, or claim that may be made by its manufacturer, is not guaranteed or endorsed by the publisher.
