# Corrigendum: Modeled energetics of bacterial communities in ancient subzero brines

**DOI:** 10.3389/fmicb.2023.1355342

**Published:** 2024-01-10

**Authors:** Georges Kanaan, Tori M. Hoehler, Go Iwahana, Jody W. Deming

**Affiliations:** ^1^School of Oceanography and Astrobiology Program, University of Washington, Seattle, WA, United States; ^2^NASA Ames Research Center, Moffett Field, CA, United States; ^3^International Arctic Research Center, University of Alaska Fairbanks, Fairbanks, AK, United States

**Keywords:** cryopeg, Arctic, extremophiles, permafrost, maintenance energy

In the published article, there was an error. The temperature of the permafrost is incorrectly characterized as near-constant; although temperature was always below freezing, some changes occurred during the period considered.

A correction has been made to Material and Methods, Model assumptions and limitations, 2. This sentence previously stated:

“The assumption is not unreasonable given the hydrological isolation of the brines and the near-constant temperatures that have kept their surroundings frozen throughout their lifetimes (Iwahana et al., 2021; Osman et al., 2021).”

The corrected sentence appears below:

“The assumption is not unreasonable given the hydrological isolation of the brines and the temperatures that have kept their surroundings frozen throughout their lifetimes (Iwahana et al., 2021; Osman et al., 2021).”

There was another error resulting from a typo. The stated exponent for the value of dissolved inorganic carbon in CBIW is incorrect because the multiplier 10 was typed twice.

A correction has been made to Results, Brine dissolved inorganic carbon, 1. This sentence previously stated:

“The dissolved inorganic carbon content of frozen sediment from CBIW (in 2018) was 4.9 × 10 × 10^10^ fg C mL−1”.

The corrected sentence appears below:

“The dissolved inorganic carbon content of frozen sediment from CBIW (in 2018) was 4.9 × 10^10^ fg C mL−1”.

The authors apologize for these errors and state that they do not change the scientific conclusions of the article in any way. The original article has been updated.

