# Supplementary material for: Modeled energetics of bacterial communities in ancient subzero brines
Source: Front Microbiol. 2023 Jul 26;14:1206641. doi: 10.3389/fmicb.2023.1206641 (PMC10411740; doi:10.3389/fmicb.2023.1206641)
Supplement: Supplementary file 1 [file Data_Sheet_1.docx]

Supplementary Material

Modeled energetics of bacterial communities in ancient subzero brines

Georges Kanaan^*^, Tori M. Hoehler, Go Iwahana, Jody W. Deming

*** Correspondence:** Georges Kanaan: georges@gkanaan.com

**
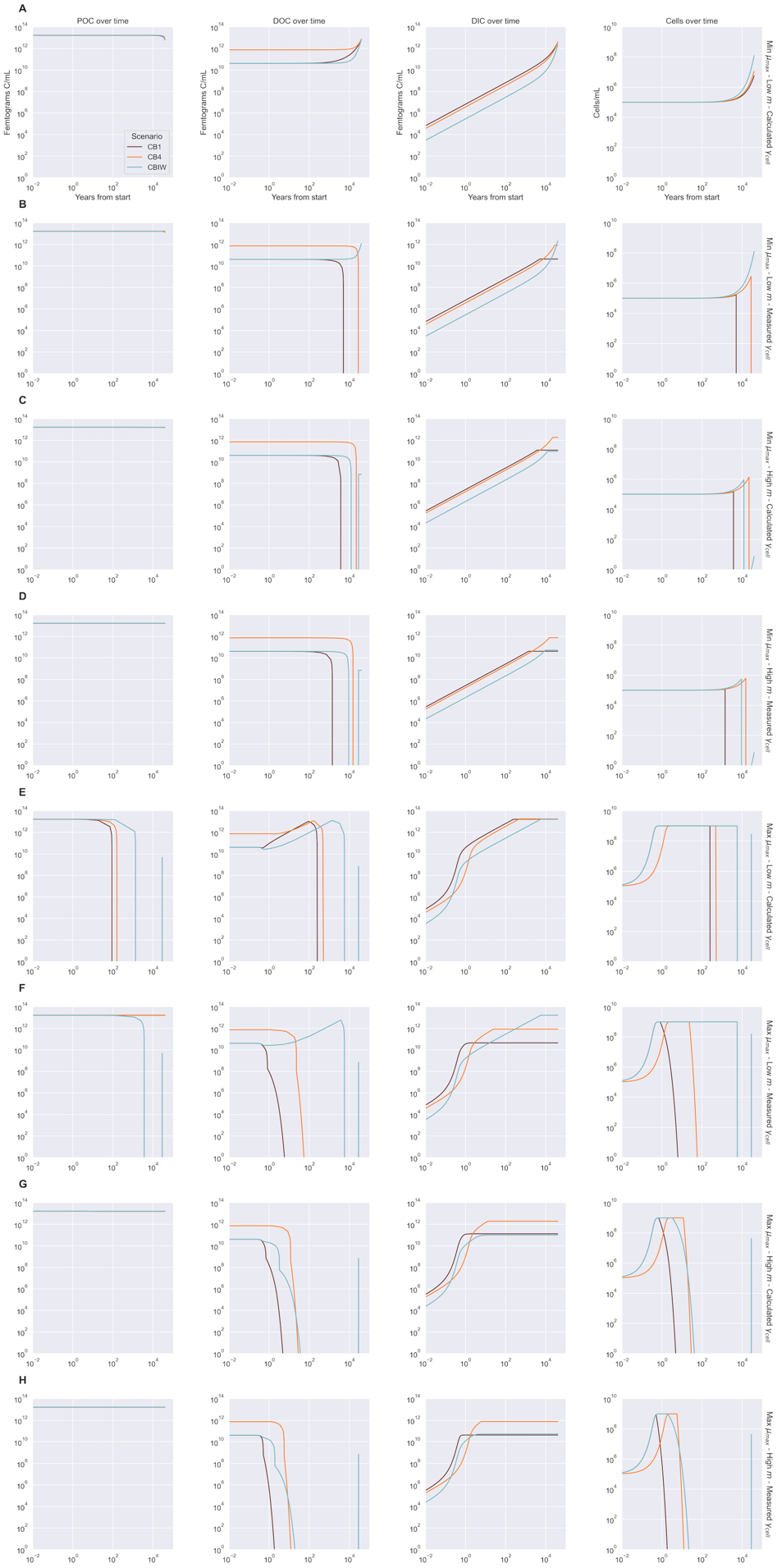
**

**Figure S1.** Model predictions of particulate (POC) and dissolved organic carbon (DOC), dissolved inorganic carbon (DIC), and cell density over the lifetime of the system for each cryopeg brine scenario (CB1, CB4, CBIW). Each row (A–H) depicts simulations based on a unique combination of conditions: minimum or maximum growth rate (µ_max_), low or high cell-specific metabolic rate (*m*), and measured or calculated cell-specific extracellular enzyme activity (EEA) rate ($\alpha_{cell}$). The resulting variations show the importance of EEA, which dictates the quantity of available DOC to the system. When EEA is high enough to allow the community to use all available POC, as in row E, the energetic demand posed by the fast-growing community depletes the available organic system in the system before simulation end. In other cases, such as in row G, significant quantities of POC remain due to an EEA bottleneck.
